# Protocol methodology for permission release in the construction of a written corpus

**DOI:** 10.1016/j.mex.2022.101754

**Published:** 2022-06-09

**Authors:** Muhamad Fadzllah Zaini, Mazura Mastura Muhammad, Norliza Jamaluddin, Md. Zahril Nizam Md. Yusoff, Nordiana Hamzah, Noor Zuhidayah Muhd Zulkifli, Mohd Haniff Mohd Tahir, Sharmini Pillai

**Affiliations:** a Department of Malay Language and Literature, Faculty of Languages and Communication, Universiti Pendidikan Sultan Idris, 35900, Tg. Malim, Perak, Malaysia; b Department of English Language and Literature, Faculty of Languages and Communication, Universiti Pendidikan Sultan Idris, 35900, Tg. Malim, Perak, Malaysia; c Industrial Law Journal, 59000, Bangsar, Selangor, Malaysia

**Keywords:** Copyright, Corpus linguistics, Written data

## Abstract

Copyright issues or permission releases for written data remain vague and difficult to comprehend. Overall, the construction of a corpus involves a multitude of mediums, namely books, periodicals, published materials and non-published materials. Problems arise when a chosen medium involves published materials that are protected under copyright acts. Therefore, this article highlights a protocol for obtaining permission release on written data to facilitate the construction of a corpus. This protocol was designed based on the concept of BNC corpus construction and adapted to the Malaysian copyright protocol framework (MyIPO). The construction of this protocol serves as a source of reference for data ethics at the Corpus and Forensic Linguistics Unit, Faculty of Languages and Communication, which is located at Sultan Idris Education University. The findings of this protocol highlighted nine pertinent steps for releasing copyright permission for written corpus. The implication of establishing this protocol are first, researchers are able to utilize textual data without forfeiting the copyright acts; second, owners of creations are protected from exploitation and finally, legal practitioners are able to enforce the owners’ copyright appropriately.

Overall, this methodology:

• Facilitates the permission release process for any copyrighted written data;
• Assists and preserves copyright laws, which allow written data to be used only through the proper channels;
• Protects researchers from any legal implications related to copyright acts concerning written data.

## Graphical abstract

**Figure:**
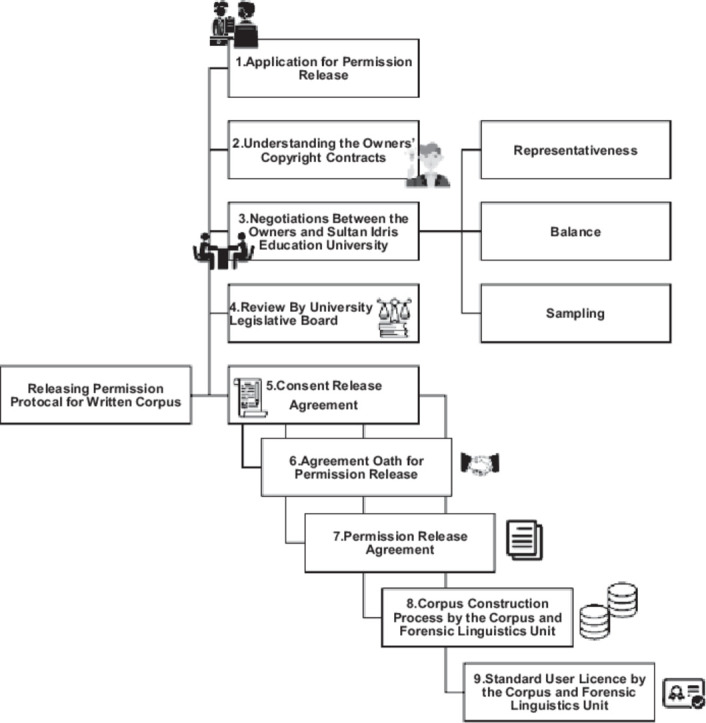

Specifications table

**Table:** 

Subject Area:	Computer Science
More specific subject area:	*Corpus Linguistics*
Method name:	*Protocol for Obtaining Permission Release On Written Data*
Name and reference of original method:	• *Reference Guide for the British National Corpus (XML Edition) edited by Lou Burnard, February 2007. URL:*http://www.natcorp.ox.ac.uk/XMLedition/URG/ • *Copyright Licensing Body. Retrieved September 8, 2021, from Intellectual Property Corporation of Malaysia (MyIPO) website:*https://www.myipo.gov.my/en/copyright-licensing-body/
Resource availability:	*N/A*

## Method overview

The release of copyright permission on written data is a persistent legal issue among academics. Most linguists use written texts as their principal source of research data. As such, it is essential to develop the correct protocol for releasing the copyright permission for these written texts. This is pertinent to avoid any disputes between authors, publishers and researchers. In Malaysia, MyIPO is referred to as the agency that protects copyright legally and the Copyright Act 1987 states that *“...users (individuals/ organizations with business premises) who intend to use any copyrighted work owned by a person must first obtain permission from the owner of the work...”* [6]. The construction of a corpus must abide by the copyright act of the nation where it is established [5]. Hence, researchers need to be vigilant and careful when engaging with the copyright release protocol.

**Fig. 1:**
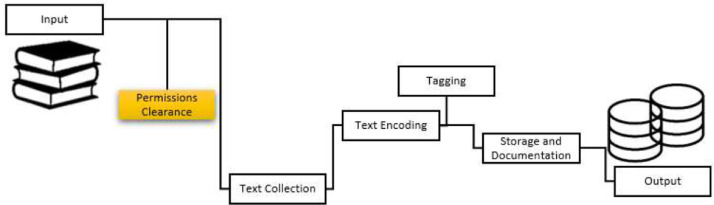
The methodology for constructing a reference written corpus (Adapted from the British National Corpus).

The principle design behind this reference corpus construction derived from the standards set during the development of the British National Corpus (BNC), which began in 1991. Fig. 1 shows five main priorities that are determined in constructing a reference corpus, namely permission release, corpus collection, corpus encoding, corpus annotation and storage and documentation [4,8]. In the current study, these priorities are observed to ensure that standardized data is collected, which will facilitate the use of data for any global users [3,5]. The construction of such a protocol helps to avoid any legal consideration bias [1]. In short, once permission to use the texts has been obtained, the texts could be included in the corpus (the text is converted into plain text files (.txt)). These texts are then converted into a standard project coding format and sorted by file names [9]. Next, the texts are annotated automatically for speech word tagging (POS-Tagging). Upon completion, these files are rearranged and included in the corpus. Each level of corpus processing is recorded in the corpus construction at the Corpus and Forensic Linguistics Unit, Faculty of Languages and Communication, Sultan Idris Education University, Tanjong Malim, 35900, Perak, Malaysia.

### Protocol for permission release

At the beginning of the Project, and following consultation with various authors, publishers and other interested parties, a standard procedure was decided on. This approach was necessary because obtaining permission release for all the texts used in the corpus can be costly, particularly since the construction of the corpus involves thousands of copyrighted texts.

The important features of the standard procedure are listed below:

• The copyright owner agrees to allow their materials to be included without licensing charges and, in return, the researchers agree that the corpus will not be commercially exploited;
• The researchers agree that a copy of the corpus will only be provided based on the conditions of the standard licence agreement;
• The researchers agree that the texts included in the corpus will not be the full texts;
• A standard licence for the users is generated based on this approval and agreed upon by all copyright owners.

Table 1 shows the nine steps for implementing the permission release protocol for the use of text material as corpus data. Adherence to this protocol allows the owner and the user to negotiate and reach an agreement (a win situation). This protocol enables researchers to use the textual data without infringing the copyright acts and reminds researchers to always be careful and respect the owner's creation. In ensuring that this action is carried out continuously, the owner, researcher and University Legislative Board should come to an agreement prior to construction of the written corpus. The average user or researcher was previously unaware of the binding nature or application of the copyright acts of each country. This legislation protects the owner's creation from misuse and exploitation by others. During the implementation of this protocol, several aspects must be considered by the researcher (the Corpus Unit) to obtain permission. Table 2 outlines the parallel steps that could be applied during the negotiations between the owner and Sultan Idris Education University (refer to Step 3 in Table 1).

**Table 1:** The nine steps releasing permission protocol for written corpus.

	Steps	Outcomes	Methods	Officers Involved
Releasing Permission Protocol for Written Corpus	***1. Application for Permission Release***	Obtain metadata of documents containing copyright.	Analysis of the metadata classification of the copyrighted documents. Identify the owners and contracts.	Officer at the Corpus and Forensic Linguistics Unit Owner
	***2. Understanding the Owners’ Copyright Contracts***	Obtain information from the owners and understand their rights. (Optional - Determine whether the owners have contracts with second or third parties).	Meet the owners to review the copyright contracts.	Officer at the Corpus and Forensic Linguistics Unit Legal officer
	***3. Negotiations Between the Owners and Sultan Idris Education University***	Written agreement between the owners and Sultan Idris Education University.	Meet for negotiations and agreement between the owners and Sultan Idris Education University.	Officer at the Corpus and Forensic Linguistics Unit Legal officer
	***4. Review by University Legislative Board***	Review or change the Consultation/ Agreement by the University Legislative Board.	All consultations and agreement documentation must be subject to a review process conducted by the University Legislative Board.	Officer at the Corpus and Forensic Linguistics Unit Owner Legal officer
	***5. Consent Release Agreement***	Written consent for permission release.	Meet the owners to obtain signatures of authorization for release agreement.	Officer at the Corpus and Forensic Linguistics Unit Owner Legal officer
	***6. Agreement Oath for Permission Release***	Documentation Agreement with Commissioners for Oaths Council.	Meet to obtain signature of authorization from the Commissioners for Oaths Council for release agreement.	Officer at the Corpus and Forensic Linguistics Unit Owner Legal Officer at the Commissioners for Oaths Council
	***7. Permission Release Agreement***	The documentation of the permission release agreement.	Submit the documentation authorizing the release agreement.	Officer at the Corpus and Forensic Linguistics Unit Owner Legal officer
	***8. Corpus Construction Process by* the Corpus and Forensic Linguistics Unit**	The construction of written corpus.	Construct the written corpus based on the corpus construction methodological framework, as stated by Crawford and Csomay [2].	Officer at the Corpus and Forensic Linguistics Unit
	***9. Standard User Licence by* the Corpus and Forensic Linguistics Unit**	Standard User Licence by the Corpus Unit.	Once the construction of the corpus is complete, licences are issued to users.	Officer at the Corpus and Forensic Linguistics Unit

**Table 2:** Parallel steps for negotiations between the copyright owners and Sultan Idris Education University.

	Steps	Outcomes	Methods
Parallel Steps	***Representativeness***	General and specialized corpora	Determine the language, register, domains, genre, dates, authors and region.
	***Balance***	Corpus Size	Determine the targeted total word count, which contains the classification percentage by domains, dates, mediums, authors, regions, readers and the required features.
	***Sampling***	Population, sampling unit, sampling frame	Text chunks are sampled using the initial, middle or end chunks of each text.

Table 2 displays the important parallel steps that highlight the precise procedure for obtaining permission release. These steps enhance the overall understanding of how written data is obtained. Along with representative data, balanced and sample data are used. The written data is not used in full in the corpus construction. Instead, only selected samples are chosen, such as the beginning, middle and end of the texts [7]. Copyright owners and researchers from the Corpus and Forensic Linguistics Unit should use this procedure as a means to increase their research endeavours in the education sphere and the higher learning sector. The design of the protocol not only allows the resolution of issues concerning copyright acts but also means the user demonstrates their appreciation to the owner while conducting research. Owners will realize the importance of copyrighted documents and how these documents can be utilized for educational and research purposes. The researcher's role is to respect and use the correct legal channels, according to the copyright acts, to obtain permission from the owners.

## Conclusion

This paper presents and shares the nine steps protocol for permission release of written data, such as books and materials of a written nature containing copyright. This protocol involves nine core steps for requesting permission release. In addition, three parallel steps are highlighted in ensuring the owner understands how their creation will used for research purposes. Written data was not obtained in full; instead, only samples were utilized in the corpus construction. Hence, the measures used can address the needs of a nation and its legislation. Proposals for future protocols could examine the construction of a spoken corpus, which would require a set of procedures to obtain verbal data from individuals from different demographic parameters. The procedures and protocol designed for this purpose would facilitate the preservation and documentation of spoken language. It is recommended that future researchers submit protocols that recognize the value of authentic results and documentation. It is also suggested that the nine steps protocol should be adopted by other units that are directly involved in corpus construction or development bearing in mind the Copyright Act of each nation.
